# Diagnostic delay in women with cancer: What do we know and which factors contribute?

**DOI:** 10.1016/j.breast.2025.104427

**Published:** 2025-02-19

**Authors:** Liza A. Hoveling, Melinda Schuurman, Sabine Siesling, Kristel M. van Asselt, Christina Bode

**Affiliations:** a Department of Health Technology and Services Research, Technical Medical Centre, University of Twente, Enschede, the Netherlands; b Department of Research and Development, Netherlands Comprehensive Cancer Organisation (IKNL), Utrecht, the Netherlands; c Department of Health Sciences, Faculty of Sciences, VU University Amsterdam and Amsterdam Public Health Research Institute, Amsterdam, the Netherlands; d Department of General Practice, Amsterdam UMC, University of Amsterdam, Amsterdam, the Netherlands; e Department of Psychology, Health and Technology, University of Twente, Enschede, the Netherlands

**Keywords:** Diagnostic delays, Breast cancer, Individual-level factors, Bronfenbrenner's ecological model, High-income countries

## Abstract

Timely cancer diagnosis is important, but delays are common, also among women. This study reviews recent literature on diagnostic delays in women with breast cancer, focusing on individual-level factors and their interaction with micro, meso, exo, and macrosystem factors. Following PRISMA-ScR guidelines, we conducted a scoping review on diagnostic delays in cancer among women, including qualitative and quantitative studies with oncological patients or healthcare professionals. We searched PubMed/MEDLINE and Scopus for publications from 2018 to November 28, 2023, excluding studies not meeting the inclusion criteria, not in English or Dutch, or focused solely on cancer screening. Titles and full texts were screened, with disagreements resolved through discussion. Two reviewers independently extracted study details, population characteristics, study design, and factors contributing to diagnostic delays. Initially, 9699 records were retrieved, resulting in 129 relevant studies after exclusions. We focused on women's health and breast cancer, narrowing our scope to 22 studies in high-income countries. Studies explored diagnostic delays and factors at various levels: microsystem (demographics, health behaviours, psychology, healthcare interactions), mesosystem (schedules, peer and support networks), exosystem (social, cultural, environmental, accessibility factors), and macrosystem (broader cultural, societal contexts, healthcare policies). In high-income countries, diagnostic delays in breast cancer care involve factors across various systems, affecting individuals, peers, healthcare, and policies. Enhancing awareness, communication, and access is important, requiring targeted campaigns and infrastructure upgrades. The Bronfenbrenner's ecological model effectively addresses the multifaceted factors influencing diagnostic delays. Future research can benefit from applying this model to various cancers and income settings.

## Highlights

Study Highlights.

1. **Focus**: Diagnostic delays in breast cancer among women in high-income countries, analyzed using Bronfenbrenner's ecological model.
2 **Key Findings**:
  • **Microsystem:** Psychological barriers (fear, denial), low income, and poor health behaviors increase delays. Solutions include education, counselling, and better healthcare access.
  • **Mesosystem:** Social support reduces delays, while work and family duties cause postponements. Community-based interventions can help.
  • **Exosystem:** Immigration status, ethnicity, and underinsurance prolong delays. Recommendations include improved care access and provider communication.
  • **Macrosystem:** Stigma, discrimination, and policy gaps hinder timely diagnosis. Suggested actions include anti-stigma campaigns and expanded screening programs.
3 **Conclusion**: A holistic, policy-driven approach is essential. Future research should address gender disparities and include diverse income settings.

## 1 Introduction

Timely cancer diagnosis is important for better outcomes, as prognosis depends on the stage at diagnosis [1]. Early diagnosis improves cancer management and outcomes by reducing delays between symptom recognition, presentation, and diagnosis [[1], [2], [3], [4], [5]]. Delays lead to higher cancer stages, lower survival rates, higher healthcare costs, and negative impact on patients' quality of life and satisfaction [6,7]. Research in the Netherlands shows disparities in diagnostic timelines [[8], [9], [10]], with the patient interval, from symptom recognition to visiting a GP, being the longest [10]. Around 10–25 % of cancer patients in the Netherlands face prolonged diagnostic intervals [8]. Gender may also play a role, as women often wait for seeking help or receive alternative diagnoses more frequently than men, affecting timely diagnosis [9,[11], [12], [13], [14]]. Healthcare professionals may also prioritise alternative diagnoses over cancer, especially in women, whose somatic symptoms are often less specific or unexplained compared to men [[14], [15], [16], [17], [18]]. Despite some progress, addressing persistent gender-based inequalities is important for better cancer outcomes and societal health [[19], [20], [21]].

Determinants of diagnostic delay can be examined using an ecological model, which highlights various layers of influence on behaviour. This model could help to explore how factors at different levels (micro-, meso-, exo- and macrosystem level) influencing the individual (e.g., individual-level factors) contribute to delays in cancer diagnosis. Studies on cancers in both men and women have identified contributing factors at multiple levels. At the microsystem level, individual behavioural factors such as the frequency of doctor visits and lifestyle habits like smoking can impact diagnostic delays [13,[22], [23], [24], [25], [26]]. Emotional dimensions, including coping mechanisms, anxiety levels, and health hardiness, are integral in understanding how individuals respond to potential symptoms of cancer [25,[27], [28], [29], [30]]. Cognitive factors such as knowledge, awareness, risk perceptions, and personal beliefs about health play a pivotal role in shaping how quickly individuals seek medical help [22,[29], [30], [31], [32]]. Within the mesosystem level, the interconnections between different microsystems become relevant. Social factors such as competing responsibilities (e.g., childcare, work commitments) and the presence of support networks influence when and how individuals seek medical advice [23,25,29]. The interaction between personal life and healthcare access is important in determining diagnostic delays. The exosystem level encompasses broader contexts that indirectly affect the individual, such as demographic aspects. Factors like employment status, living arrangements, income, educational attainment, age, and marital status influence access to and utilization of healthcare services [[11], [12], [13],[23], [24], [25],^28^,^29^,[32], [33], [34], [35], [36], [37]]. These elements shape the external environment, impacting healthcare accessibility and decision-making. At the macrosystem level, societal norms, cultural values, and economic conditions influence individual behaviours and attitudes towards health. These broader societal influences set the context within which individuals make health-related decisions [38].

In this study we review recent literature on diagnostic delays in women, focusing on individual-level factors and their interaction with micro, meso, exo, and macrosystem factors. Moreover, to effectively classify findings in literature, we used Bronfenbrenner's ecological model [39] which addresses how factors at various levels influencing the individual (e.g., individual-level factors). Importantly, our innovative approach to organize and interpret information on delay systematically through the application of Bronfenbrenner's ecological model allows to integrate delay factors on different socio-cultural levels. Our research is especially important for women, as they face unique barriers to timely diagnosis, including societal norms, gendered communication patterns, and biases in medical decision-making. These factors often lead to delays that disproportionately affect their prognosis and quality of life. By addressing challenges of women, we aim to provide evidence for gender-specific interventions.

## 2 Methods

To comprehensively assess the scope of publications on diagnostic delay in women with cancer, before focusing specifically on breast cancer, we initially conducted a broad scoping review of studies on diagnostic delay in cancer among women. This review was carried out in accordance with the Preferred Reporting Items for Systematic Reviews and Meta-Analyses for Scoping Reviews (PRISMA-ScR) guidelines [40,41]. Detailed descriptions of the review methodology are provided in the following sections.

### 2.1 Literature search strategy

PubMed/MEDLINE and Scopus databases were searched separately for relevant studies using the search queries detailed in Table 1 (and Supplementary Tables 1 and 2). The protocol was registered online at Open Science Framework (OSF) registries (https://osf.io/pwgt8). To identify potential studies related to diagnostic delay, we incorporated relevant free-text terms, which were specified for article titles exclusively due to their broad scope in abstract searches. Applied search strategy included relevant oncological terms (e.g., cancer, oncology, neoplasm and carcinoma) and terms associated with diagnostic delay (e.g., delay, postponed, disrupted). Databases and search strategies were reviewed in collaboration with information specialists, and pilot testing was conducted to ensure that all studies previously identified by the authors were included. The final database searches were executed on 28th November 2023.

**Table 1:** Search queries used for titles, abstracts, and keywords in the database search performed on 28th November 2023.

Database	Query
PubMed/MEDLINE	(("diagnos∗"[Title/Abstract] AND "delay∗"[Title/Abstract]) OR ("care"[Title/Abstract] AND "delay∗"[Title/Abstract]) OR "patient delay"[Title/Abstract] OR "presentation delay∗"[Title/Abstract] OR "timely diagnos∗"[Title/Abstract] OR "primary care delay∗"[Title/Abstract] OR "late diagnos∗"[Title/Abstract]) AND ("cancer"[Title/Abstract] OR "tumor"[Title/Abstract] OR "neoplasm"[Title/Abstract] OR "malignan∗"[Title/Abstract] OR "carcino∗"[Title/Abstract] OR "oncolog∗"[Title/Abstract] OR "sarcoma"[Title/Abstract] OR "leukemia"[Title/Abstract] OR "lymphoma"[Title/Abstract] OR "melanoma"[Title/Abstract] OR "blastoma"[Title/Abstract]) AND ("determinant∗"[Title/Abstract] OR "influence∗"[Title/Abstract] OR "barrier∗"[Title/Abstract] OR "factor∗"[Title/Abstract] OR "reason∗"[Title/Abstract]) AND ("women"[Title/Abstract] OR "woman"[Title/Abstract] OR "female"[Title/Abstract] OR "gender"[Title/Abstract] OR "sex"[Title/Abstract] OR "breast"[Title/Abstract] OR "cervi∗"[Title/Abstract] OR "uter∗"[Title/Abstract] OR "endometr∗"[Title/Abstract] OR "ovar∗"[Title/Abstract] OR "vulv∗"[Title/Abstract])
Scopus	(TITLE-ABS-KEY((diagnosis AND delay) OR (care AND delay) OR "patient delay" OR "presentation delay" OR "timely diagnosis" OR "primary care delay" OR "late diagnosis")) AND (TITLE-ABS-KEY(cancer OR tumor OR neoplasm OR malignant OR carcinoma OR oncology OR sarcoma OR leukemia OR lymphoma OR melanoma OR blastoma)) AND (TITLE-ABS-KEY (determinant OR influence OR barrier OR factor OR reason)) AND (TITLE-ABS-KEY (women OR woman OR female OR gender OR sex OR breast OR cervix OR uterus OR endometrium OR ovary OR vulva))

### 2.2 Inclusion and exclusion criteria

Qualitative and quantitative studies meeting the inclusion criteria encompassing oncological patients or healthcare professionals working with oncological patients, patient-related factors, along with data pertaining to cancer patients, their behaviours, cognitive aspects, emotional responses, and diagnostic processes conducted by healthcare professionals, were considered eligible for analysis. Studies failing to meet these criteria, not available in English or Dutch or focusing only on screening were excluded. Furthermore, studies falling under categories other than "original research", such as “systematic review”, "commentary," "letter to the editor," or "editorial," were also excluded. To ensure focus in the review and select the most recent studies, we decided to concentrate on studies conducted between 2018 and 2023.

### 2.3 Study screening

The titles of all identified studies were initially screened by L.H. after removing duplicates. Subsequently, L.H. screened the full texts of the included studies. Concurrently, M.S. reviewed the titles and full texts of 20 % of all identified records between years 2018–2023. Reviewers reached agreement over 86 % of these studies, and the remaining 14 % were discussed by L.H. and M.S. In the event of disagreement, both reviewers discussed which studies to include. Six of these studies (13 %) were further examined by C.B., K.A., and S.S.

### 2.4 Data extraction

Data extraction from the studies was conducted by L.H., with M.S. independently extracting data from 20 % of the studies. Prior to analysis, consensus was reached between the two extractors. The extracted information from each study included: the last name of the first author, year of publication, country, GDP per capita, classification of the country's income level (low, middle, or high), method of cancer detection, study type (qualitative or quantitative), gender composition of the study population, type of cancer investigated, study objectives, characteristics of the study population, study center, year of population inclusion, study design, type of delay assessed, individual factors examined, and the individual-level factors identified.

### 2.5 Consultation

As an addition to the PRISMA-ScR checklist, the methodological framework of Arksey and O'Malley [41] proposes to add consultation. Therefore, meetings for consultations were performed. In these meetings, preliminary results were presented, and the interdisciplinary author team, consisting of a GP, a psychologist, and an epidemiologist, was asked to provide input. The input was processed to get to the results presented below.

### 2.6 Data analysis

After carefully screening publications across all ecological levels, we discovered sufficient literature to proceed with classifying factors using Bronfenbrenner's ecological model. This model categorises and groups individual-level factors by delineating the layers that shape an individual's behaviour and experiences [39]. By applying this framework to the findings, we explored how various individual-level factors interact within their environment, alongside external influences and broader cultural and societal norms. Given that breast cancer is a disease that predominantly affects women, gender differences in diagnostic delays are obviously relevant. However, the focus on breast cancer in this study limits the ability to directly compare gendered factors, as breast cancer is not a disease experienced by men with the same incidence or in the same context. Therefore, while gender-related disparities in healthcare exist, the unique nature of breast cancer means that gender differences are not pronounced in our analysis. Our approach provided valuable insights into the complexities of diagnostic delays in breast cancer among women.

## 3 Results

### 3.1 Search results

**Fig. 1:**
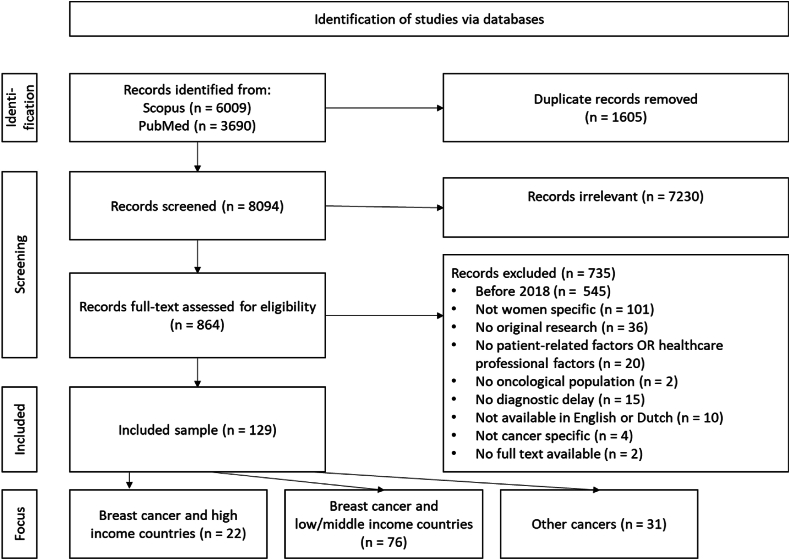
PRISMA flowchart depicting the process of study selection and the rationale for exclusions during full-text screening.

The database search retrieved a total of 9699 records, with 1605 duplicates being excluded. Within the remaining set of 8094 unique records, 7230 were excluded following title screening in accordance with the exclusion criteria. Fig. 1 provides a flow diagram depicting the records that were discovered, screened, selected, and subsequently excluded along with the corresponding exclusion criteria. The remaining 129 studies underwent full-text examination.

### 3.2 To bring focus

**Fig. 2:**
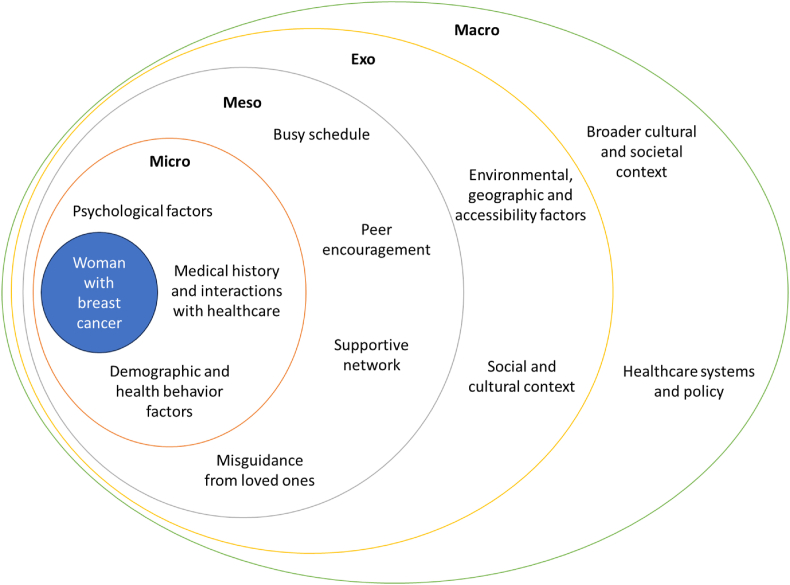
Ecological system of woman with breast cancer and diagnostic delay.

After reviewing 129 studies and considering the methodology of our scoping review, we determined that refining our approach would enhance precision and coherence in the results section, despite the extensive literature on women's health and female cancers. Our aim was not to fragment the dataset, but rather to establish a focused analytical framework. Therefore, in this review, we examine 22 studies that specifically investigate diagnostic delays among women diagnosed with breast cancer in high-income countries [42], elucidating the interplay of individual-level factors influencing such delays. Breast cancer, the most common cancer in women worldwide [43]. One in eight women will face breast cancer in their lifetime [44]. While not fully preventable, reducing risk factors can lower morbidity and mortality [45,46]. Early detection is important to avoid advanced disease, invasive treatments, and lower survival rates [47,48]. Breast cancer often presents with palpable symptoms, making it important to study diagnostic delays in this context, as these delays lead to poorer outcomes. Screening programs increase early detection but have limited attendance and age coverage [49]. Applying the Bronfenbrenner ecological model to the findings, various individual-level factors interact within their environment, alongside external influences and broader cultural and societal norms were categorised (Fig. 2 & Supplementary Table 3).

### 3.3 General overview of included studies

A summary of the 22 included studies on individual-level factors of diagnostic delay is presented in Supplementary Table 4 [[50], [51], [52], [53], [54], [55], [56], [57], [58], [59], [60], [61], [62], [63], [64], [65], [66], [67], [68], [69], [70], [71]]. Most studies (n = 10) were conducted in the United States [53,55,57,59,60,[62], [63], [64],^67^,^72^]. Symptomatic detection of breast cancer was predominant in 12 studies [51,53,56,61,[63], [64], [65],^68^,^70^,^71^,^73^,^74^]. Quantitative methodologies were used in 17 studies [51,54,55,57,[59], [60], [61], [62], [63], [64], [65],^67^,^68^,[70], [71], [72],^74^], with nine focusing on symptomatically detected breast cancer [51,61,[63], [64], [65],^68^,^70^,^71^,^74^].

#### 3.3.1 Microsystem

Investigating microsystem factors that impact diagnostic delays in breast cancer diagnosis was the focus of twenty studies [51,52,[54], [55], [56], [57],[59], [60], [61], [62], [63], [64], [65], [66], [67], [68],^70^,^71^,^73^,^74^]. These studies dissected the factors contributing to diagnostic delay, three distinct subcategories were divided.

Psychological factors have been identified as influences on the delay in diagnosing breast cancer. Quantitative studies consistently point to denial, fear of potential cancer diagnosis, anxiety about medical findings, and general apprehension regarding medical consultations as contributors to prolonged diagnostic intervals [51,55,74]. However, some quantitative research suggests that self-doubt about initial symptoms and external locus of control and perceived health competence might decrease delays, indicating that heightened awareness and proactive symptom recognition can expedite the diagnostic process [68,71]. Qualitative research has further enriched these insights by highlighting the complexities of emotional barriers that contribute to diagnostic delays. These include fear of cancer or medical procedures, anxiety, symptom exacerbation, denial, aversion to medical tests, fear of healthcare providers, and fatalistic attitudes influenced by cultural norms such as machismo (e.g., expecting symptoms to resolve on their own) [52,56,66,73]. Studies also indicate that knowledge of breast cancer symptoms correlates with decreased delay [71]. Moreover, qualitative studies emphasise the role of coping strategies, belief systems, risk perceptions, misinterpretation of symptoms, and stoicism in shaping individuals' responses to symptoms and subsequent healthcare-seeking behaviours [52,56,66,73]. Individuals' diverse perceptions of symptoms—such as changes in initial presentation, perceived stability, fluctuations, urgency, and normalization—also play an important role in their decisions regarding seeking healthcare and can result in diagnostic delays [52,53,56,73].

Demographic and health behaviour factors exerted varying degrees of influence on diagnostic delay. Pain, higher income and higher educational level were identified as influential in decreasing delay, being postpartum increases delay while age exhibited heterogeneous effects across studies [59,[61], [62], [63],^68^,^70^,^71^]. While self-healthcare practices (e.g., breast massage and taking traditional Chinese medicine) were associated with decreased diagnosis time in breast cancer, life style behaviours such as smoking addiction were linked to increased delays [68]. Qualitative studies further emphasised the importance of proactive symptom management, monitoring, alternative medicine usage, and the influence of cultural beliefs on health-related decision-making processes to reduce diagnostic timelines [52,73].

For medical history and interactions with healthcare, major and minor comorbidities were found to be associated with prolonged diagnostic intervals [70]. On the other hand, individuals in good health, as well as those with a medical or family history of cancer, experienced shorter diagnostic timelines [[59], [60], [61]]. A factor such as symptom disclosure emerged as decreasing diagnostic time, while barriers such as being too busy to seek help and language barriers were identified as increasing delays [57,61,74]. Qualitative research added factors such as medical history of breast cancer, adherence to routine breast examinations, previous healthcare experiences, and continuity of care have emerged as determinants of diagnostic timelines. Furthermore, the importance of transparent communication, established relationships with healthcare providers, preferences for specific practitioners, and the influential role of social support networks have been highlighted in increasing the diagnostic process [52,56,66].

#### 3.3.2 Mesosystem

Exploring mesosystem factors that contribute to diagnostic delays in breast cancer diagnosis was the primary focus of one quantitative and four qualitative studies [52,56,66,67,71,73]. Social support emerged as important factor for decreasing diagnostic delay [67,71]. Among the factors identified in qualitative studies, encouragement from friends or family emerged as increases in individuals' healthcare-seeking behaviours [56]. Positive reinforcement and supportive networks were found to encourage individuals to prioritise their health concerns and seek timely medical attention, potentially reducing diagnostic delays. Conversely, false reassurance or incorrect advice from family members or friends was highlighted as a barrier to prompt diagnosis [73]. Misinformation or dismissive attitudes from close contacts could lead individuals to downplay the significance of their symptoms or delay seeking medical help, ultimately prolonging the time to diagnosis. Competing personal commitments (related to work or family) were also identified as an important factor contributing to diagnostic delays within the mesosystem context [52,56,66,73].

#### 3.3.3 Exosystem

Examining exosystem factors that impact diagnostic delays in breast cancer diagnosis was the focus of eighteen studies [51,52,[54], [55], [56], [57],^59^,^60^,[62], [63], [64], [65], [66], [67], [68],^70^,^72^,^73^]. These studies analyzed the web of external influences shaping individuals' healthcare-seeking behaviours and diagnostic timelines. To provide an understanding of these influences, we subdivided the exosystem factors into two distinct categories.

Foremost among the exosystem factors influencing delay were social and cultural contexts. Having an immigration status, having an Hispanic ethnicity (compared to non-Hispanic), and being underinsured emerged as determinants of longer diagnostic timelines [54,57,70]. Certain other subpopulations faced heightened diagnostic delays. For instance, black sexual minority women, sexual minorities in general, and individuals from Black and low-income communities in high income countries experienced prolonged diagnostic intervals [55,59,60,64,67].

Environmental, geographical, and accessibility factors influence diagnostic delays. Here, challenges in timely appointments with healthcare providers and disparities in access to care based on residential location were identified as contributors to diagnostic delays [61,72,74]. Qualitative studies shed light on a spectrum of variables, ranging from the holiday period to urbanicity levels and living conditions, each playing an inhibiting role in shaping individuals' healthcare-seeking behaviours. Moreover, logistical challenges such as scheduling constraints, extended waiting times for preferred specialists, the unavailability of regular general practitioners, and the commute duration from residence to hospital emerged as factors hindering timely access to diagnosis [52,56,66,73].

#### 3.3.4 Macrosystem

Exploring macrosystem factors that influence diagnostic delays in breast cancer diagnosis was the focus of fourteen studies [52,53,56,60,61,[63], [64], [65], [66], [67], [68],^70^,^72^,^74^].

At the forefront of broader cultural and societal context factors was the influence of stigma (e.g., experiences of stigma measured by the Intersectional Discrimination Index-Major scale), which emerged as an inhibitor to diagnostic timelines. For instance, participants reported instances of being refused care by healthcare providers, evicted or denied housing, and other discriminatory experiences based on their identities [67]. Qualitative studies underscored the detrimental effects of stigma on help-seeking behaviours, social sanctioning, and overall awareness of breast cancer, highlighting the role in prolonging diagnostic intervals [52,56,66].

Healthcare systems and policy, emerged as a determinant of delay. Healthcare utilization was identified as a double-edged sword, with increased utilization paradoxically associated with prolonged diagnostic timelines in some contexts [68,70]. Moreover, qualitative insights shed light on various systemic barriers such as lack of telehealth accessibility and financial constraints all of which increased diagnostic delays [52,56,66].

## 4 Discussion

In our scoping review, we initially focused on diagnostic delays and individual-level factors across all cancer types. However, we found that the majority of studies centred on women with breast cancer. Therefore, we narrowed our focus to diagnostic delays and individual-level factors specifically in women with breast cancer from high income countries. We extracted findings from 22 studies and organised them effectively using Bronfenbrenner's ecological model [39], identifying various individual-level factors.

### 4.1 Microsystem

In our study, psychological factors like denial and fear, alongside demographic influences such as low income and unhealthy behaviours, increased delay times. Additionally, medical history and healthcare interactions highlighted the impact of comorbidities and effective symptom communication on diagnostic timelines. Based on our findings, several intervention strategies can be proposed to address diagnostic delays in breast cancer. Educational initiatives are important to enhance awareness of symptoms and reduce barriers to early medical consultation [75]. Psychosocial support, including counselling, could help address anxiety and denial, which often contribute to delayed diagnoses. Improving access to healthcare through expedited referrals and reduced wait times can shorten the interval between symptom presentation and diagnosis [76]. Additionally, culturally sensitive interventions are needed to address diverse perceptions and attitudes towards health and medical care [77]. Healthcare providers could also adopt diagnostic checklists to ensure that key symptoms are systematically investigated, minimizing missed opportunities for early detection. Additionally, tailored interventions should address psychological barriers, such as fear and denial, through culturally relevant educational materials that are tailored to diverse patient groups (among women).

### 4.2 Mesosystem

We found social support of closed others to play an important role in reducing delays. Positive reinforcement and supportive networks encouraged individuals to prioritise their health concerns and seek timely medical attention. Conversely, misinformation or dismissive attitudes from close contacts could lead to delayed diagnosis. Competing personal commitments, such as work or family responsibilities, also contributed to delays. Research shows that support from family and friends often reduces fear, stigma, and misinformation. These factors frequently delay women from seeking timely medical help for breast cancer symptoms [78,79]. Another study pointed out that women who received encouragement from their social circles were more likely to recognize symptoms and promptly seek medical advice, thereby reducing the interval between symptom onset and diagnosis [80]. Interventions targeting these factors could involve promoting social support from family and friends, decreasing fear and misinformation, and dispelling misconceptions through targeted public campaigns. Moreover, interventions that leverage familial and community networks to provide culturally sensitive education and support can further enhance these efforts. These interventions can address specific social perceptions and attitudes towards healthcare, ensuring that women receive the necessary encouragement and information to pursue timely diagnostic evaluations [79,80]. Employers and community leaders can also play a role by promoting flexible work hours and community support programs that reduce competing responsibilities and encourage timely health-seeking behaviour. Workplace campaigns could highlight the importance of early diagnosis and provide resources for women balancing work and caregiving roles.

### 4.3 Exosystem

Social and cultural contexts affected delay times, we found in our study, with factors like immigration status, Hispanic ethnicity, and underinsurance associated with longer diagnostic intervals. Subpopulations such as black sexual minority women and individuals from low-income communities in high income countries also experienced prolonged delays. Environmental and accessibility factors, including challenges in securing timely appointments and disparities based on residential location, are also important. Logistical barriers such as scheduling constraints and long waits for specialist care further hindered timely access to diagnosis. Recent studies highlight the important role of healthcare providers in reducing diagnostic delays for women with breast cancer. One systematic review found that failure to follow up on abnormal mammograms is an issue, with various factors such as lack of timely communication and inadequate follow-up plans contributing to delays. Healthcare providers play an important role in addressing these gaps by ensuring that patients are informed promptly about abnormal results and by establishing clear follow-up protocols [81]. Another study, although in a low income country, emphasised that logistical barriers within health service delivery, such as long waiting times for appointments and test results, impact diagnostic intervals. Effective strategies include enhancing provider communication, ensuring timely follow-ups on abnormal mammograms, and improving referral systems [82,83]. Another strategy could involve utilizing support from patient organizations, where patients can find information about the physical symptoms they are experiencing. Healthcare providers can decrease these delays by streamlining referral processes and improving access to diagnostic services. For instance, direct referrals to specialised care and bypassing secondary care facilities have been shown to expedite the diagnostic process [84]. Furthermore, healthcare providers' attitudes and behaviours influence the diagnostic journey. Provider training to improve communication skills and cultural competency can enhance patient trust and adherence to follow-up recommendations, thereby reducing delays [85]. Healthcare systems should establish programs to assist patients in overcoming logistical and administrative hurdles, such as scheduling appointments or accessing specialised care. These programs could particularly benefit underinsured populations or individuals from marginalized communities.

### 4.4 Macrosystem

In our review, broader cultural and societal context, encompassing pervasive stigma such as experiences of discrimination and social sanctioning, impeded timely diagnosis. Stigmas impact on help-seeking behaviours and awareness of breast cancer, prolong delays. Healthcare system dynamics and policies played a role. Systemic barriers such as limited telehealth access and financial constraints further contributed to delays. Recent studies show telehealth improves early disease detection, offering convenience and enabling better patient counselling and medication management. However, challenges remain, including the inability to conduct physical examinations, technology barriers for vulnerable patients, and potential impacts on physician-patient relationships [86]. Patient's fitness, stress levels, and non-verbal cues like anxiety, nervousness, and physical condition cannot be observed. Interventions should target stigma reduction through anti-discrimination initiatives, improve healthcare accessibility via telehealth enhancements, and advocate for policies supporting timely diagnosis and treatment. Telehealth has expanded healthcare access, benefiting rural and mobility-limited populations. Implementing policies that expand implementation of screening programs, increase funding for mammography, and reduce barriers to seeking care is important. Research has shown that countries with screening programs and accessible diagnostic facilities (e.g., mobile mammography unit) have more effective breast cancer management than countries without proper facilities [[87], [88], [89]]. Policymakers should prioritise funding for mobile diagnostic units and public awareness campaigns aimed at reducing stigma. These campaigns should address both cultural perceptions of cancer and practical barriers, such as fear of financial costs. Enhanced training for healthcare providers to recognize diagnostic cues in women can also improve early detection rates.

### 4.5 Strengths and limitations

A notable strength of this study is the use of Bronfenbrenner's ecological model to organize results, allowing for a clear and comprehensive presentation of the individual-level factors influencing diagnostic delays in breast cancer among women. Another strength is the methodology of the review, the diverse expertise of the authors, including a GP, a psychologist, and two epidemiologists, provided varied perspectives. A specialised librarian ensured the protocol and search strategy were thorough. Two independent reviewers assessed the studies with high agreement, resolving any discrepancies through discussion, and additional reviewers examined a subset of studies, further enhancing the rigor of the study. While this scoping review offers valuable insights into individual-level factors affecting diagnostic delays in breast cancer care, we have to acknowledge several limitations. The reliance on existing literature introduces potential selection bias due to variations in methodology and study quality. Although efforts were made to decrease this bias through search strategies and strict inclusion criteria, the lack of (behavioural) models used in the studies may still pose a challenge. Another limitation of our study is the lack of available research on individual-level factors influencing diagnostic delays in general cancer types between men and women. To maintain focus, we specifically concentrated on breast cancer in high-income countries, excluding studies from low- and middle-income countries and other types of cancer.

## 5 Conclusion

Diagnostic delays in cancer between men and women are understudied, particularly regarding individual-level factors. By focusing on breast cancer care among women in high-income countries, we found that diagnostic delays are influenced by multifaceted factors across micro, meso, exo, and macro systems, including individuals, their social circles, healthcare providers, and policy influences. Improving symptom awareness, fostering open communication about symptoms, and addressing healthcare accessibility are important steps. Actionable strategies are needed, such as implementing patient navigation programs and diagnostic checklists to address barriers within healthcare systems. Policymakers should focus on expanding access to mobile mammography units and subsidizing diagnostic procedures to ensure equity in care. Moreover, tailored public awareness campaigns targeting underserved communities are important to decrease stigma and misinformation. A holistic approach that integrates medical and policy considerations is important to address diagnostic delays. Tailoring interventions to individual beliefs, behaviours, culture and preferences is likely more effective than standardised methods. Challenges in reaching underserved populations highlight the importance of involving patient groups in shaping interventions. Future research should prioritise gender-specific studies to improve early detection and outcomes for women with cancer. Diagnostic delays should also be explored between genders and in low- and middle-income countries. Our study shows that Bronfenbrenner's ecological model effectively integrates the multifaceted factors influencing diagnostic delays. Future research can benefit from applying this model to various cancers and income settings to improve diagnosis and care especially in women but also across diverse populations.

## CRediT authorship contribution statement

**Liza A. Hoveling:** Conceptualization, Data curation, Formal analysis, Investigation, Methodology, Project administration, Resources, Validation, Visualization, Writing – original draft, Writing – review & editing. **Melinda Schuurman:** Data curation, Formal analysis, Writing – review & editing. **Sabine Siesling:** Conceptualization, Funding acquisition, Methodology, Project administration, Resources, Supervision, Validation, Writing – review & editing. **Kristel M. van Asselt:** Conceptualization, Investigation, Methodology, Validation, Writing – review & editing. **Christina Bode:** Conceptualization, Data curation, Formal analysis, Funding acquisition, Investigation, Methodology, Project administration, Resources, Supervision, Validation, Writing – review & editing.

## Disclaimer

All results are available in Supplementary Table 4.

## Ethical approval

Ethical approval was not required for this study, as it is a scoping review based on publicly available data from previously published studies. No primary data collection involving human participants or personal data was conducted. The review followed established methodological guidelines, including the PRISMA-ScR framework, ensuring transparency and rigor in the research process.

## Funding

No funding was received for this study.

## Conflict of interest

The authors declare no conflict of interest.
