# Supplementary material for: Diagnostic delay in women with cancer: What do we know and which factors contribute?
Source: Breast. 2025 Feb 19;80:104427. doi: 10.1016/j.breast.2025.104427 (PMC11904510; doi:10.1016/j.breast.2025.104427)
Supplement: Multimedia component 1 [file mmc1.docx]

**Supplementary Table 1. Keyterms Pubmed**

| **#** | **Keyterm** | **Search string** | **Total hits** |
| --- | --- | --- | --- |
| 1 | Diagnostic delay | (((diagnos*[Title/Abstract]) AND (delay*[Title/Abstract])) OR ((care[Title/Abstract]) AND (delay*[Title/Abstract])) OR "patient* delay*" [Title/Abstract] OR "presentation delay*" [Title/Abstract] OR "timely diagnos*" [Title/Abstract] OR "primary care delay*" [Title/Abstract] OR "late diagnos*" [Title/Abstract]) | 158,774 |
| 2 | Cancer | (cancer [Title/Abstract] OR tumor [Title/Abstract] OR neoplasm [Title/Abstract] OR malignan* [Title/Abstract] OR carcino* [Title/Abstract] OR oncolog* [Title/Abstract] OR sarcoma [Title/Abstract] OR leukemia [Title/Abstract] OR lymphoma [Title/Abstract] OR melanoma [Title/Abstract] OR blastoma [Title/Abstract]) | 4,104,394 |
| 3 | Factors | (determinant* [Title/Abstract] OR influence* [Title/Abstract] OR barrier* [Title/Abstract] OR factor* [Title/Abstract] OR reason* [Title/Abstract]) | 6,419,852 |
| 4 | Women | (women [Title/Abstract] OR woman [Title/Abstract] OR female [Title/Abstract] OR gender [Title/Abstract] OR sex [Title/Abstract] OR breast [Title/Abstract] OR cervi* [Title/Abstract] OR uter* [Title/Abstract] OR endometr* [Title/Abstract] OR ovar* [Title/Abstract] OR vulv* [Title/Abstract]) | 3,827,663 |
| 5 |  | #1 AND #2 AND #3 AND #4 | 3,690 |

**Supplementary Table 2. Keyterms Scopus**

| **#** | **Keyterm** | **Search string** | **Total hits** |
| --- | --- | --- | --- |
| 1 | Diagnostic delay | TITLE-ABS-KEY((diagnosis AND delay) OR (care AND delay) OR "patient delay" OR "presentation delay" OR "timely diagnosis" OR "primary care delay" OR "late diagnosis") | 128,799 |
| 2 | Cancer | TITLE-ABS-KEY(cancer OR tumor OR neoplasm OR malignant OR carcinoma OR oncology OR sarcoma OR leukemia OR lymphoma OR melanoma OR blastoma) | 6,549,118 |
| 3 | Factors | TITLE-ABS-KEY(determinant OR influence OR barrier OR factor OR reason) | 16,703,664 |
| 4 | Women | TITLE-ABS-KEY(women OR woman OR female OR gender OR sex OR breast OR cervix OR uterus OR endometrium OR ovary OR vulva) | 13,510,247 |
| 5 |  | #1 AND #2 AND #3 AND #4 | 7,807 |

**Supplementary Table 3. Factors from the 22 studies categorized within Bronfenbrenner's ecological model**

| ***Microsystem*** |  |
| --- | --- |
| **Psychological factors** | Fear of cancer or procedure/disgust, anxiety, depressive symptoms, worsening of symptoms, denial, worries about the possibility of being diagnosed with breast cancer, unaware about the warning symptoms of breast cancer, feeling too embarrassed to have breast examination by the physician, worries about the treatment consequences which might include a surgical intervention, self-concern about initial symptom, feeling scared about what the doctor might find, feeling worried about what the doctor might find, fear of tests/investigations, fear of doctor response, distrust, fatalism, machismo, external health locus of control, perceived health competence, coping, beliefs, risk perception, misinterpretation of BC symptoms attributed to other diseases or expectation of more alarming/obvious BC symptoms, stoicism, initial response on first noticing possible BC symptoms, matching of knowledge to breast cancer symptoms, change in initial symptom, persistence of symptom, perceived stability of symptoms, fluctuating symptoms, sense of urgency, normalization of BC symptoms attributed to hormonal changes, dietary changes, or work-induced stress. |
| **Demographic and health behaviour** | Age, pregnant, postpartum, number of children, body mass index, cognitive impairment, pain stimulation, occupation, educational level, socioeconomic status, living below poverty line, underlying disease, marital status, unemployed seeking work, college degree, household income, self-health care, smoking, alcohol drinking habit, self-manage symptoms, monitor, wait to resolve, alternative medicine, health literacy. |
| **Medical history and interactions with healthcare** | Personal history of breast cancer, breast density, prior breast biopsy, time since last mammogram, cancer history, previous diagnosis of diabetes/hypertension, family history of BC, routine breast examination, previous experience, reasons for first medical consultation, number of medical visits before being diagnosed, major comorbidities, minor morbidities, good health, Charlson Comorbidity Score, Continuity of Care Index, detection method, disclosure of symptoms, established relationship with general practitioner, preference for female GP, reluctance to see new GP, reluctance to see bulk-billing GP, too busy to seek medical help, primary language. |
|  |  |
| ***Mesosystem*** |  |
| **Encouragement from friends or family** |  |
| **False reassurance or incorrect advice from family members or friends** |  |
| **Competing personal commitments** |  |
| **Social support** |  |
|  |  |
| ***Exosystem*** |  |
| **Social and cultural context** | Insurance, immigration status, race and ethnicity, religion, white sexual minority women, white heterosexual women, black sexual minority women, black heterosexual women, black, white, sexual minority, heterosexual, black poverty. |
| **Environmental, geographic and accessibility factors** | Holiday period, level of urbanicity, living conditions, length of time between contacting a doctor and getting an appointment, length of time between being referred to hospital and getting an appointment with a specialist, scheduling, waiting time for preferred specialist, lack of availability of regular GP, travelling time from residence to hospital, away from home, difficulty of getting an appointment with the physician, access to care, area of residence. |
|  |  |
| ***Macrosystem*** |  |
| **Broader cultural and societal context** | Social sanctioning/encouragement of help-seeking, stigma, fear from having breast cancer stigmata in society, social awareness. |
| **Healthcare systems and policy** | Telehealth, cost to see GP, distance to hospital, healthcare utilization. |

**Supplementary Table 4. Study characteristics and factors related to diagnostic delay in cancer in women**

| **Title** | **Author/year** | **Country** | **Qualitative or quantitative study** | **Aims of the study** | **Study population** | **Study center** | **Year of population inclusion** | **Study design** | **Type of delay** | **Individual factors** | **Most imporant individual factors** |
| --- | --- | --- | --- | --- | --- | --- | --- | --- | --- | --- | --- |
| Comparing Pathways to Diagnosis and Treatment for Rural and Urban Patients with Colorectal or Breast Cancer: A Qualitative Study | Bergin et al. - 2020 | Australia | Qualitative | We compared rural and urban patient experiences of pathways to colorectal or breast cancer diagnosis and treatment in Victoria, Australia. | Following procedures for the ICBP project,18 the population-based Victorian Cancer Registry sent ICBP surveys to colorectal and breast cancer patients aged 40 years or older within 6 months of diagnosis. Those with synchronous or multiple primary cancers or whose specialist advised not to approach were excluded. Specific to Victorian patients, surveys included an expression of interest form for participation in further research. | Victorian Cancer Registry | November 2013 to October 2014 | Semi-structured telephone interviews were conducted with 43 patients (49% colorectal; 60% rural, median 7 months post-diagnosis). A framework analysis was applied using the Model of Pathways to Treatment. | Patient and system-factor themes in the diagnostic interval | Symptom interpretation: nonurgent/noncancer sign, prior experience, natural cause, health lifestyle/not a candidate for cancer. Symptom attributes: nonspecific symptoms, intermittent, vague, slow change, self-manage symptoms, monitor, wait to resolve. Emotions/attitudes: embarrassment/disgust, fear of tests/investigations, fear of doctor response, irregular help-seeker, fatalism, stoicism, machismo. Scheduling and practical issues: away from home, holidays, other priorities/busy (e.g. family, work), transport, cost. Health system and clinician-related delays: lack of PCP continuity, PCP waiting time. Help-seeking triggers: symptom interpretation, change, abnormal, severity, intuition, context: social sanctioning/awareness, scheduling, health literacy | Symptom interpretation: nonurgent/noncancer sign, prior experience, natural cause, health lifestyle/not a candidate for cancer. Symptom attributes: nonspecific symptoms, intermittent, vague, slow change. Scheduling and practical issues: away from home, holidays, other priorities/busy (e.g. family, work). Health system and clinician-related delays: PCP waiting time. Help-seeking triggers: symptom interpretation, change, abnormal, severity, intuition, context: social sanctioning/awareness. |
| Patients' help-seeking experiences and delaying in breast cancer diagnosis: A qualitative study | Oshiro et al. - 2018 | Japan | Qualitative | To identify the influencing factors in help-seeking behavior by comparing delayers with non-delayers in Japanese female patients with breast cancer. | A total of 21 female patients with breast cancer (nine delayers and 12 non-delayers) who were at least 3 months to 5 years postdiagnosis were recruited from two hospitals in Okinawa, Japan. | two hospitals in Okinawa, Japan | September and November 2015 | Semistructured interviews were carried out and the results were analyzed by using a qualitative inductive approach. | “delays” are defined as patients in which there was a prolonged interval between the time they first noticed a cancer symptom and their initial seeking of a provider evaluation | Barrier: presentation of symptoms, anxiety and fear of cancer, living conditions, disclosure of symptoms, coping, beliefs, risk perception, distance to medical care. Trigger: the presence of other persons who encourage seeking a provider evaluation, worsening of symptoms, surrounding circumstances. | The comparison between the delayers and non-delayers showed eight barriers to help-seeking behavior that were unique to the delayers: (i) the appearance of symptoms that cannot be definitely linked to breast cancer; (ii) anxiety and fear; (iii) the necessity to prioritize the immediate needs of daily life; (iv) non-disclosure of the situation; (v) the view that medical care is a nuisance; (vi) a desire to surrender to the natural course of things; (vii) confidence that they would not develop cancer; and (viii) inaccessibility of medical facilities. A common trigger for help-seeking that was identified in both the delayers and the nondelayers was the presence of other persons who encouraged seeking a provider evaluation. |
| Factors influencing early presentation to general practitioners for non–screen detected breast cancer | Foo and Rhee - 2022 | New South Wales | Qualitative | This study aimed to identify and explore the differences in help-seeking behaviours between rural and urban women with non–screen-detected breast cancer | We conducted a qualitative study consisting of semistructured interviews with 20 women from New South Wales with non–screen detected breast cancer that was diagnosed within the past five years. | Modified Monash in New South Wales | April to June 2021 | We conducted a qualitative study consisting of semistructured interviews with 20 women from New South Wales with non–screen detected breast cancer that was diagnosed within the past five years. | Patient delay - help seeking delay | Perception of breast cancer risk, History of benign breast conditions, Other plausible cause to explain symptom, Matching of knowledge to breast cancer symptoms, Change in initial symptom, Persistence of symptom, Perceived stability of symptoms, Fluctuating symptoms, Sense of urgency, Denial, Stigma, Stoicism, Encouragement from friends or family, Social awareness, Competing personal commitments, Holiday period, COVID-19, Established relationship with general practitioner (GP), Previous negative experience, Concerns about unnecessary presentation, Preference for female GP, Reluctance to see new GP, Reluctance to see bulk-billing GP, Telehealth, Lack of availability of regular GP, Cost to see GP, Distance to GP | There was little difference between rural and urban groups in terms of symptom appraisal and monitoring, social interactions, and personal and environmental factors. However, the presence of stoicism as a barrier was unique to rural women. Rural women also faced significant barriers in accessing general practice services that pertained to availability, cost and distance. |
| Exploring causes of delays in help-seeking behaviours among symptomatic Omani women diagnoses with late-stage breast cancer - A qualitative study | Al-Azri et al. - 2022 | Oman | Qualitative | This study aimed to explore causes of delays in medical help-seeking behaviours among symptomatic Omani women diagnosed with late-stage BC. | This study was conducted at the Sultan Qaboos University Hospital (SQUH) and Royal Hospital (RH), the two main oncological referral centres in the country. These hospitals are located in Muscat, the capital city of Oman. Both SQUH and RH provide comprehensive oncological treatment for cancer patients, including those diagnosed with BC. All women diagnosed with BC at other hospitals in Oman, regardless of region, are referred to either RH or SQUH for surgical and oncological treatment. | This study was conducted at the Sultan Qaboos University Hospital (SQUH) and Royal Hospital (RH), the two main oncological referral centres in the country. |  | Purposeful sampling was used to identify Omani women diagnosed with late-stage BC (i.e., stages III or IV) at the two main referral oncology centres in Oman. Semi-structured individual interviews were utilised to collect data regarding the participants’ reasons for delaying seeking medical help. The qualitative framework analysis approach was used for data analysis. | Delays in medical help-seeking behaviours | Six reasons for delays in seeking medical help were identified, including: (1) Being in denial of BC symptoms; (2) normalisation of BC symptoms attributed to hormonal changes, dietary changes, or work-induced stress; (3) misinterpretation of BC symptoms attributed to other diseases or expectation of more alarming/ obvious BC symptoms; (4) pursuit of alternative medicine remedies; (5) false reassurance or incorrect advice from family members or friends; and (6) practical barriers, such as childcare responsibilities and lack of access to transport. | Six reasons for delays in seeking medical help were identified, including: (1) Being in denial of BC symptoms; (2) normalisation of BC symptoms attributed to hormonal changes, dietary changes, or work-induced stress; (3) misinterpretation of BC symptoms attributed to other diseases or expectation of more alarming/ obvious BC symptoms; (4) pursuit of alternative medicine remedies; (5) false reassurance or incorrect advice from family members or friends; and (6) practical barriers, such as childcare responsibilities and lack of access to transport. |
| Identification of barriers at the primary care provider level to improve inflammatory breast cancer diagnosis and management | Devi et al. - 2023 | USA | Qualitative + Quantitative | The purpose of this study, based in the United States, was to evaluate knowledge gaps and barriers related to diagnosis and care of inflammatory breast cancer (IBC), a rare but lethal breast cancer subtype, amongst Primary Care Providers (PCP) as they are often the first point of contact when patients notice initial symptoms. | PCP participants in the Duke University Health System, federally qualified health center, corporate employee health and community practices, nearby academic medical center, Duke physician assistant and advanced practice nurse leadership program alumni were first selected in a convenience sample and for semi-structured interviews (n =11). | PCP participants in the Duke University Health System, federally qualified health center, corporate employee health and community practices, nearby academic medical center, Duke physician assistant and advanced practice nurse leadership program alumni were first selected in a convenience sample and for semi-structured interviews (n =11). | Between August 2020 and April 2021 | Between August 2020 and April 2021, experienced graduate degree trained qualitative interviewers and data analysts (LJF, MF) from the Duke Cancer Institute Behavioral Health and Survey Research core (BHSRC) conducted semi-structured interviews with 11 PCPs via Zoom. | Diagnostic delay | Barriers to diagnosis, barriers to care, health disparities, explaining inflammatory breast cancer to patients, referrals and connecting to cancer centers, educational strategies | PCP reported access to care and knowledge gaps in symptom recognition (mean =3.3, range 1–7) as major barriers. Only 31 % reported ever suspecting IBC in a patient. PCP (n = 49) responded being challenged with referral delays in diagnostic imaging. Additionally, since the COVID-19 pandemic started, 63 % reported breast cancer referral delays, and 33 % reported diagnosing less breast cancer. PCP stated interest in CME in their practice for improved diagnosis and patient care, which included online (53 %), lunch time or other in-service training (33 %), patient and provider-facing websites (32 %). |
| Patient-Reported Experiences of Breast Cancer Screening, Diagnosis, and Treatment Delay, and Telemedicine Adoption during COVID-19 | Du et al. - 2022 | United States | Quantitative | To evaluate and quantify potential sociodemographic disparities in breast cancer screening, diagnosis, and treatment due to the COVID-19 pandemic, and the use of telemedicine. | Individuals aged 18 or older were eligible for the survey if they were: (1) receiving—or planning to receive—routine screening mammograms; (2) undergoing diagnostic evaluation for breast cancer; or (3) had ever been diagnosed with breast cancer. | one of the five collaborating breast cancer advocacy organizations (Dr. Susan Love Foundation for Breast Cancer Research (West Hollywood, CA, USA), SHARE Cancer Support (New York, NY, USA), SurvivingBreastCancer.org (Boston, MA, USA), Sisters Network Inc. (Houston, TX, USA), and TOUCH, The Black Breast Cancer Alliance (Annapolis, MD, USA)), or through invitations sent to women age 40–74 using ResearchMatch. | 14 May and 1 July 2020 | This cross-sectional web-based survey was administered between 14 May and 1 July 2020. Data were collected via REDCap, a secure online survey software program [23]. Participants accessed and completed the survey either by emailing the research team and receiving a private survey link or by clicking a public link distributed via social media by the study team through advertisements by one of the five collaborating breast cancer advocacy organizations | Screening, Diagnosis, and Treatment Delay | Personal history of breast cancer, age, race and ethnicity, U.S. Region, level of urbanicity, healthcare site, college degree, household income, insurance | Insurance (medicaid causes delay compared to private insurance) |
| Delays in Breast cancer care by race and sexual orientation: results from a national survey with diverse women in the united states | Poteat et al. - 2021 | USA | Quantitative | This study sought to understand delays in breast cancer care by examining the intersection of race and sexual orientation. | Participants met the following inclusion criteria: 35 years of age or older; United States resident; assigned female at birth and identified as a woman; history of breast cancer within the prior 10 years or an abnormal clinical breast exam, mammogram, ultrasound, or MRI within the prior 24 months. We aimed to enroll at least 100 each of the following groups: Black sexual minority women, Black heterosexual women, White sexual minority women, and White heterosexual women. | We used a community-engaged participatory approach to this anonymous online crosssectional study. ZAMI NOBLA – National Organization of Black Lesbians on Aging (ZAMI NOBLA) collaborated with academic researchers in the design and implementation of the study as well as interpretation of study findings. | Accrual began in July 2018 and closed to all except Black sexual minority women in September 2018. | This online cross-sectional survey enrolled racially and sexually diverse women age ≥ 35 years who had been diagnosed with breast cancer within the prior 10 years or had an abnormal screening in the prior 24 months. | Care delay | White sexual minority women, white hetero sexual women, black sexual minority women, white hetero sexual women, black, white, sexual minority, heterosexual, age, college education, income, unemployed seeking work, health insurance, social support score, mean stigma score, charlson comorbidity score | Black SMW (n=101) had the highest prevalence of care delays with a 5.17-fold increased odds of care delay compared with White heterosexual women (n=298) in multivariable models. Black SMW reported higher intersectional stigma and lower social support than all other groups. In models adjusted for race, sexual orientation, and income, intersectional stigma was associated with a 2.43-fold increase in care delay; and social support was associated with a 32% decrease in odds of care delay. |
| The Effect of Hospital Characteristics on Delays in Breast Cancer Diagnosis in Appalachian Communities: A Population-based Study | Louis et al. - 2018 | USA | Quantitative | The objective was to examine whether certain characteristics of the hospital where breast cancer wasdiagnosed—its ownership structure (eg, public or private), specialized capabilities, system membership, and size—were related to delays in diagnosis for patients living in Appalachian communities. | Study data were derived from the Kentucky, North Carolina, Ohio, and Pennsylvania state central cancer registries (2006-2008). We then linked Medicare enrollment files and claims data (2005-2009), the Area Resource File (2006-2008), and the American Hospital Association Annual Survey of Hospitals (2007) to create an integrated data set. | the Kentucky, North Carolina, Ohio, and Pennsylvania state central cancer registries (2006-2008). | We then linked Medicare enrollment files and claims data (2005-2009), the Area Resource File (2006-2008), and the American Hospital Association Annual Survey of Hospitals (2007) to create an integrated data set. | Hierarchical linear modeling was used to regress the natural log of breast cancer diagnosis delay on a number of hospital-level, demographic, and clinical characteristics. | Diagnostic delay | Demograhic characteristics: race, age, marital status, diagnosis year, diagnosis quarter, diagnosis state, patient ZIP code of residence at diagnosis date, median income in county of patient residence. Tumor/cancer characteristics: cancer stage at diagnosis, lymph node involvement, hormone receptor status. Morbidity indicators: number of comorbid conditions. | Age older than 75 years |
| Breast Cancer Diagnosis, Treatment, and Outcomes of Patients From Sex and Gender Minority Groups | Eckhert et al. - 2023 | USA | Quantitative | To evaluate the quality of breast cancer treatment and recurrence outcomes for patients from sex and gender minority groups compared with cisgender heterosexual patients. | Oncoshare integrates information from the EMRs of 2 San Francisco Bay Area healthcare systems, Stanford University Health Care and Sutter Medica lNetwork, with patient-level data from the California Cancer Registry, which comprise registries that are also part of the Surveillance, Epidemiology, and End Results Program. | Oncoshare integrates information from the EMRs of 2 San Francisco Bay Area healthcare systems, Stanford University Health Care and Sutter Medica lNetwork, with patient-level data from the California Cancer Registry, which comprise registries that are also part of the Surveillance, Epidemiology, and End Results Program. | between January 1, 2008, and January 1, 2022 | In this case-control study, a keyword search algorithm was used to identify patients from SGM groups treated at Stanford University between January 1, 2008, and January 1, 2022, with an International Statistical Classification of Diseases and Related Health Problems, Tenth Revision (ICD-10) diagnosis of a breast neoplasm and the presence of a SOGI identifier, as has been described previously for cohort identification of SGM patients with cancer. | Diagnostic delay | Age at diagnosis, year of diagnosis, race and ethnicity, neighborhood SES, private insurance, SGM identity or behavior | Compared with cisgender heterosexual patients, those from SGM groups experienced a delay in time from symptom onset to diagnosis (median time to diagnosis, 34 vs 64 days), were more likely to decline an oncologist-recommended treatment modality (35 [38%] vs 18 [20%]). |
| Delays in Diagnosis and Treatment of Breast Cancer: A Safety-Net Population Profile | Jaiswal et al. - 2018 | Verenigde Staten | Quantitative | Given disparities in breast cancer outcomes based on socioeconomic status, we examined time to diagnosis and treatment in a safety-net hospital. | Our initial cohort consisted of all patients who received any care for breast cancer (N=120) from July 1, 2010, through June 30, 2012. Patients were identified by the hospital-based cancer registry. We excluded patients with primary stage IV disease or missing staging data (n=11) because they have a different treatment trajectory than those with stage 0–III disease. We also excluded patients with recurrent breast cancer within 5 years of primary diagnosis (n=2) because of the possibility that they were already under the care of an oncologist, and therefore their timeliness of care was not comparable to those who were newly diagnosed. An additional 2 patients who had both stage IV disease and recurrent cancer were excluded. The remaining 105 patients comprised our analytic study sample. | Denver Health and Hospital Authority (DH) | July 1, 2010, through June 30, 2012 | We conducted a retrospective cohort analysis of patients receiving breast cancer care at Denver Health and Hospital Authority (DH), which is an integrated, safety-net hospital system that serves Denver and the surrounding counties. | Diagnostic delay | Age, race/ethnicity, primary language, insurance status at time of diagnosis, Charlson comorbidity index, symptomatic at presentation, disease stage at presentation, surgical management | Factors significantly associated with longer intervals than median time included stage, method of presentation, language, surgical treatment, insurance, and ethnicity. |
| Factors associated with waiting time to breast cancer diagnosis among symptomatic breast cancer patients: a population-based study from Ontario, Canada | Webber et al. - 2021 | Canada | Quantitative | This study evaluated potential determinants of a long diagnostic interval among symptomatic breast cancer patients. | This was a population-based retrospective cohort study of all breast cancer patients diagnosed in Ontario, Canada between January 1, 2007 and December 31, 2015. | We used population-based administrative databases from ICES (previously known as the Institute for Clinical Evaluative Sciences). ICES is an independent, non-profit research institute whose legal status under Ontario’s health information privacy law allows it to collect and analyze health care and demographic data, without consent, for health system evaluation and improvement. | January 1, 2007 and December 31, 2015 | This was a population-based, cross-sectional study of symptomatic breast cancer patients diagnosed in Ontario, Canada from 2007 to 2015 using administrative health data. | The diagnostic interval was defined as the time from the earliest breast cancer-related healthcare encounter before diagnosis to the diagnosis date. | Age, number of major comorbidities, number of minor comorbidities, income quintile, rural residence, recent immigration status, living arrangement, stage at diagnosis, place of first presentation, number of healthcare encounters, Continuity of Care Index | Longer diagnostic intervals were observed in younger patients, patients with higher burden of comorbid disease, recent immigrants to Canada, and patients with higher healthcare utilization prior to their diagnostic interval. Shorter intervals were observed in patients residing in long-term care facilities, patients with late stage disease, and patients who initially presented in an emergency department. |
| Patient delay and associated factors among Chinese women with breast cancer: a cross-sectional study | Zhang et al. - 2019 | China | Quantitative | To examine the current situation of patient delay and to identify factors associated with patient delay among women with breast cancer in China. | a total of 312 Chinese women with a histologically confirmed diagnosis of breast cancer were recruited by a convenience sampling strategy from January 2017 to June 2017. | A general hospitals and one tertiary grade A specialized cancer hospital) from five regions of central Chengdu in Sichuan Province, which is located in Southwest China: West China Hospital; Sichuan Academy of Medical Sciences & Sichuan Provincial People's Hospital; General Hospital of Chengdu Military Region; Chengdu First People's Hospital; Chengdu Second People's Hospital; The Third People's Hospital of Chengdu, and Sichuan Cancer Hospital & Institute. | January 2017 to June 2017 | A cross-sectional descriptive research design was used, and a total of 312 Chinese women with a histologically confirmed diagnosis of breast cancer were recruited by a convenience sampling strategy from January 2017 to June 2017. The participants were consecutively enrolled from seven hospitals (six tertiary grade A general hospitals and one tertiary grade A specialized cancer hospital) from five regions of central Chengdu in Sichuan Province. | Patient delay | Knowledge of breast cancer symptoms, external health locus of control, BSE/CBE, perceived health competence, family support, pain stimulation, age | Knowledge of breast cancer symptoms, external health locus of control, BSE/CBE, perceived health competence, family support, pain stimulation, age |
| Factors Influencing Total Delay of Breast Cancer in Northeast of China | Ren et al. - 2022 | China | Quantitative | This study aimed to investigate the factors associated with the comprehensive delay behaviors and to evaluate its effect on outcomes in patients with breast cancer in Dalian, a northeast city of China. | This cross-sectional study was conducted by data of patients diagnosed with breast cancers in the second hospital of Dalian Medical University between January 1, 2012, and December 31, 2012. | the second hospital of Dalian Medical University | January 1, 2012, and December 31, 2012 | A retrospective chart review was conducted using a cancer registry dataset including 298 patients. | Diagnostic delay | Age at presentation, area of residence, marital status, occupation, education level, insurance, income level, initial visiting hospital level, self-concern about initial symptom, smoking, alcohol drinking habit, self-health care | Patients’ marital status, income levels, smoking status, initial visiting hospital level, self-health care, and self-concern about initial symptom were identified as the independent predictors of the total delay. |
| Enhancing social support and knowledge perception decreases patient delay in breast cancer | Li et al. - 2021 | China | Quantitative | The real factors in patient delay (divided into appraisal delay and utilization delay) for women with BC urgently need to be objectively analyzed for preventing the progression of this disease. | Mandarin-speaking or Sichuanese dialect-speaking women, aged ≥18 years, lucid, with normal comprehension and verbal expression, were recruited in the breast specialist outpatient clinics of Sichuan Cancer Hospital & Institute, Sichuan Cancer Center, School of Medicine, University of Electronic Science and Technology of China, Chengdu. | outpatient clinics of Sichuan Cancer Hospital & Institute, Sichuan Cancer Center, School of Medicine, University of Electronic Science and Technology of China, Chengdu. | April 20, 2014, and June 20, 2019 | While awaiting their diagnostic confirmation in breast clinics, the recruited women were interviewed and asked to fill in the questionnaires after having signed informed consent. | Patient delay: divided into appraisal delay and utilization delay | Age, religion, number of children, residential address, family history of BC, routine breast examination, symptom disclosure to others, reasons for first medical consultation, hospital category for first medical consultation, examinations for first medical consultation, reasons for not obtaining further confirmation, reasons for this medical consultation, underlying disease, knowledge of BC. | Concerning the influential factors for the different phases of delay, age, residential address and symptom disclosure to others led to a decrease of appraisal and patient delay. However, reasons for first medical consultation and knowledge of BC would accelerate appraisal and patient delay. |
| Time to Cancer Diagnosis in Young Women Presenting to Surgeons with Breast-Related Symptoms: a Population-Based Cohort Study | Menes et al. - 2019 | Israel | Quantitative | We examined time to cancer diagnosis in young women presenting to surgeons with breast-related complaints. | women aged 18–44 presenting to a surgeon with breast-related complaints between 2005 and 2015 in a large health care plan (n = 157,264) | Maccabi Healthcare Services (MHS) | All first visits to a surgeon between 2005 and 2015 by women aged 18–44 years, with a breast-related visit code were included. | Population-based cohort study | Diagnostic delay | Age, district of residence, marital status, socioeconomic status, pregnant, postpartum, visit code | Symptomatic women with lower a-priori likelihood of malignancy (younger age, postpartum, or nonspecific visit code) are at significantly greater risk of delayed diagnosis of cancer. |
| Knowledge of symptoms, time to presentation and barriers to medical help-seeking among Omani women diagnosed with breast cancer: a cross-sectional study | Al-Azri et al. 2021 | Oman | Quantitative | To identify knowledge of breast cancer (BC) symptoms, time taken to consult a doctor and factors contributing to delays in medical help-seeking. | The two main teaching hospitals in Oman: Royal Hospital and Sultan Qaboos University Hospital. Adult Omani women recently diagnosed with BC. | National Oncology Centre of the Royal Hospital (RH) and the Oncology Unit of the Sultan Qaboos University Hospital (SQUH), both of which are located in Muscat, the capital city of Oman. | November 2018 and April 2019 | A cross-sectional study of Omani women diagnosed with BC. | Patient delay | Initial response on first noticing possible BC symptoms, approximate duration of symptoms before contacting a doctor, length of time between contacting a doctor and getting an appointment, number of medical visits before being diagnosed, length of time between being referred to hospital and getting an appointment with a specialist, approximate length of time between first medical appointment and diagnosis, length of time between diagnosis and first receiving treatment | Barriers to seeking medical help included feeling scared (68.9%) and worried about what the doctor might find (62.8%). |
| Factors associated with time to breast cancer diagnosis and treatment in unscreened women in Portugal | Nouws et al. - 2018 | Portugal | Quantitative | Our aim was to quantify the time intervals from first detection to diagnosis and from diagnosis to first treatment among women for whom breast cancer detection had been prompted by manifestation of symptoms or routine exams, and to identify sociodemographic and clinical characteristics of the patients associated with these time intervals. | This study was based in a prospective cohort of women with newly diagnosed breast cancer admitted to the Breast Clinic of the Portuguese Institute of Oncology of Porto, Portugal (IPO-Porto). | Breast Clinic of the Portuguese Institute of Oncology of Porto, Portugal (IPO-Porto) | January and December 2012 | Cross-sectional | Time to breast cancer diagnosis and treatment | Age, education, health literacy, marital status, occupation, distance from residence to IPO-Porto, travelling time from residence to IPO-Porto, detection method, previous diagnosis of diabetes/hypertension, body mass index categories, cancer stage, cognitive impairment, anxiety, depressive symptoms | Longer time interval: detection method |
| Low Awareness of Breast Cancer and Considerable Barriers to Early Presentation Among Saudi Women at a Primary Care Setting | Khamis Al-Khamis, 2018 | Saudi Arabia | Quantitative | To evaluate the level of breast cancer awareness and perceived barriers to seeking medical care among Saudi women attending primary care services, using an internationally validated tool. | Adult women aged 18 years and above who were seeking primary care services during February 2014. | The current study was conducted at primary health care clinics (PHCC) of King Khalid University Hospital (KKUH) located in Riyadh, Saudi Arabia. | February 2014 | Cross-sectional | Diagnostic delay - patient delay | Difficulty of getting an appointment with the physician, worries about the possibility of being diagnosed with breast cancer, too busy to seek medical help, unaware about the warning symptoms of breast cancer, feeling too embarrassed to have breast examination by the physician, worries about the treatment consequences which might inclued a surgival intervention, fear from having breast cancer stigmata in the society. | Difficulty of getting a doctor appointment, worries about the possibility of being diagnosed with breast cancer and being too busy to seek medical help. |
| Racial Disparities in Diagnostic Delay Among Women with Breast Cancer | Miller-Kleinhenz et al. - 2021 | USA | Quantitative | The aim of this study was to evaluate patient characteristics that contribute to delay in diagnosis of screen-detected cancers and the contribution of delay to tumor characteristics and BC mortality. | Three hundred sixty-two White and 368 Black women were identified who were screened and received subsequent BC diagnoses within Emory Healthcare, a part of Emory University health care system (2010-2014). | Emory Healthcare, a part of Emory University health care system | 2012-2014 | Delays leading up to a BC diagnosis were defined on the basis of dates of mammographic screening, diagnostic evaluation, and biopsy. | Diagnostic delay | Demographic characteristics: total patient population, race, age category, socioeconomic status, insurance status, geographic distance to screening facilities, marital status. Tumor characteristics: stage, grade, lymph node involvement, tumor size, subtype, ER status. | Black women and women diagnosed at later stages, with larger tumor sizes, and with triple-negative tumors were more likely to experience 45 days to diagnosis. In multivariable-adjusted models, Black women had at least a two-fold increase in the odds of delay to diagnostic evaluation, biopsy delays, and total delays 45 days compared with White women. A 1.6-fold increased odds of BC mortality was observed among women who experienced total delays 45 days compared with women without delays in diagnosis. |
| Multilevel Factors Associated With Time to Biopsy After Abnormal Screening Mammography Results by Race and Ethnicity | Lawson et al., 2022 | US | Quantitative | To evaluate individual-, neighborhood-, and health care–level factors associated with differences in time from abnormal screening to biopsy among racial and ethnic groups. | Women aged 40 to 79 years who had abnormal results in screening mammograms conducted in 109 imaging facilities across 6 US states | across 7 BCSC registries25: Carolina Mammography Registry, Kaiser Permanente Washington Registry, Metro Chicago Breast Cancer Registry, New Hampshire Mammography Network, Sacramento Area Breast Imaging Registry, San Francisco Mammography Registry, and Vermont Breast Cancer Surveillance System. | between 2009 and 2019 | Prospective cohort | Individual-, neighborhood-, and health care–level factors associated with differences in time from abnormal screening to biopsy among racial and ethnic groups. | Race and ethnicity, age at screening, first-degree family history of breast cancer, breast density, prior breast biopsy, time since last mammogram, geocoded probability of high school education, geocoded probability of college education, geocoded household income level, residence, type of screening mammogram obtained, facility with academic affiliation, facility with on-site biopsy services. | Black women were the most likely to experience delays to diagnostic resolution after adjusting for multilevel factors. |
| Gendered and Racialized Social Expectations, Barriers, and Delayed Breast Cancer Diagnosis | Kim et al. - 2018 | USA | Quantitative | The aim of this study is to investigate the impact of gendered and racialized social expectations on the recognition and seeking of help to overcome barriers to timely screening mammography, particularly among Black women, in order to inform the development of more effective patient navigation interventions for reducing breast cancer disparities. | Participants were recruited from 3 hospitals on the South Side of Chicago over a three-year period. | 3 hospitals on the South Side of Chicago |  | The likelihood of obtaining a follow-up screening mammogram was compared between women who identified ≥1 barriers and those who did not. | Diagnostic delay | Black women, distrust, age, living below poverty line, high school education, cancer history, good health, black poverty | Of the 3754 women who received the Patient Navigation in Medically Underserved Areas navigation intervention, approximately 14% identified ≥ 1 barriers, which led to additional navigator contacts. Consequently, those women who reported barriers were more likely to obtain a subsequent screening mammogram. Black women, women living in poverty, and women with a higher level of distrust were less likely to report barriers. |
| The current state of timeliness in the breast cancer diagnosis journey: abnormal screening to biopsy | Vijayaraghavan et al. - 2023 | USA | Quantitative | In this study, we review the timeline of care at our facility over a 1-year period (October 2021- September 2022) and compare them with those reported by National Quality Measures for Breast Centers (NQMBC). | From the electronic medical records, we collected the total number of screens, diagnostic studies, ultrasound, and stereotactic guided biopsies for the year 2021. | Our combined annual screening workload exceeds 50,000 studies. Our academic and 3 of our community sites are designated Breast Imaging Centers of Excellence (BICOE) by the ACR. Our university facility, where the Comprehensive Breast Clinic is located is accredited by the American College of Surgeons asNAPBC(National Accreditation Program for Breast Centers) accredited. | The year 2021 | In this study, we review the timeline of care at our facility over a 1-year period (October 2021- September 2022) and compare them with those reported by National Quality Measures for Breast Centers (NQMBC). | Diagnostic delay | Race, ethnicity, location, and type of facility affect the outcome of care and contribute to delays in providing care. | Race, ethnicity, location, and type of facility affect the outcome of care and contribute to delays in providing care. |
